# Reactive oxygen species induction by cabazitaxel through inhibiting Sestrin-3 in castration resistant prostate cancer

**DOI:** 10.18632/oncotarget.21147

**Published:** 2017-09-21

**Authors:** Takeo Kosaka, Hiroshi Hongo, Yasumasa Miyazaki, Koshiro Nishimoto, Akira Miyajima, Mototsugu Oya

**Affiliations:** ^1^ Department of Urology, Keio University School of Medicine, Tokyo, Japan; ^2^ Department of Uro-Oncology, Saitama Medical University International Medical Center, Hidaka, Japan; ^3^ Department of Urology, Tokai University School of Medicine, Hiratsuka-shi, Japan

**Keywords:** castration-resistant prostate cancer, cabazitaxel, ROS, SESN3

## Abstract

Reactive oxygen species (ROS) production induced by taxanes in cancer cells may influence the taxane-induced cell death or the drug resistance. We investigated the correlation between the cytotoxic effect of taxanes and ROS production in human castration-resistant prostate cancer (CRPC) cell lines. Three human prostate cancer cell lines were treated with increasing concentrations of docetaxel or cabazitaxel *in vitro*. Cabazitaxel showed significantly higher cytotoxic efficacy than docetaxel in human CRPC cells, accompanied by elevated ROS production detected by FACS analysis. To investigate whether cabazitaxel-mediated cell death was caused by the ROS generation induced by cabazitaxel, we treated CRPC cells in the presence of antioxidant NAC. NAC reduced the cytotoxic effect induced by cabazitaxel. We found that ROS elimination by Sestrin-3 (*SESN3*) was significantly inhibited by cabazitaxel, but not by docetaxel. These results indicate higher sensitivity of human CRPC to cabazitaxel compared to docetaxel involves ROS production through inhibiting the expression of antioxidant enzyme SESN3.

## INTRODUCTION

Prostate cancer (PCa) is one of the most commonly diagnosed malignant tumors in men and the second leading cause of cancer-related deaths in Western countries [1]. One of the most troublesome aspects of PCa is that androgen-dependent PCa inevitably progresses to highly aggressive and life-threatening castration-resistant prostate cancer (CRPC) after androgen ablation therapy [2, 3]. Docetaxel has been approved by the U.S. Food and Drug Administration, leading to regulatory approval of this cytotoxic drug to treat patients with CRPC [3, 4]. Recently, cabazitaxel became the most effective cytotoxic agent to demonstrate an improvement in survival in men with docetaxel-refractory CRPC [5]. Cabazitaxel is a semisyntheric taxane that was selected for development on the basis of its poor affinity for ATP-dependent drug efflux pump P-glycoprotein compared with docetaxel and paclitaxel [6, 7]. Cabazitaxel showed activity in both docetaxel-sensitive and docetaxel-resistant cancers in preclinical testing and in clinical trials, providing the rationale for further clinical development in cancers such as metastatic CRPC [5].

Recent studies shed light on the reactive oxygen species (ROS) production induced by anticancer drugs in cancer cells, which may influence the cell death or the drug resistance [8–15]. ROS are generated by oxidative stress and sometimes associated with the sensitivity or resistance to anticancer drugs [8, 11–14, 16]. It has been reported that reduction of ROS production can modulate the cytotoxic effect of taxanes in cancer cells [13, 15, 17–19]. However, the ROS production by cabazitaxel or the mechanistic action has not characterized yet in cancer cells, including CRPC.

The objectives of the present study were to determine whether cabazitaxel could induce ROS production in CRPC and to evaluate the possible contribution of ROS in its cytotoxic effect.

## RESULTS

### Cabazitaxel can overcome docetaxel resistance in C4-2AT6 cells

**Figure 1:**
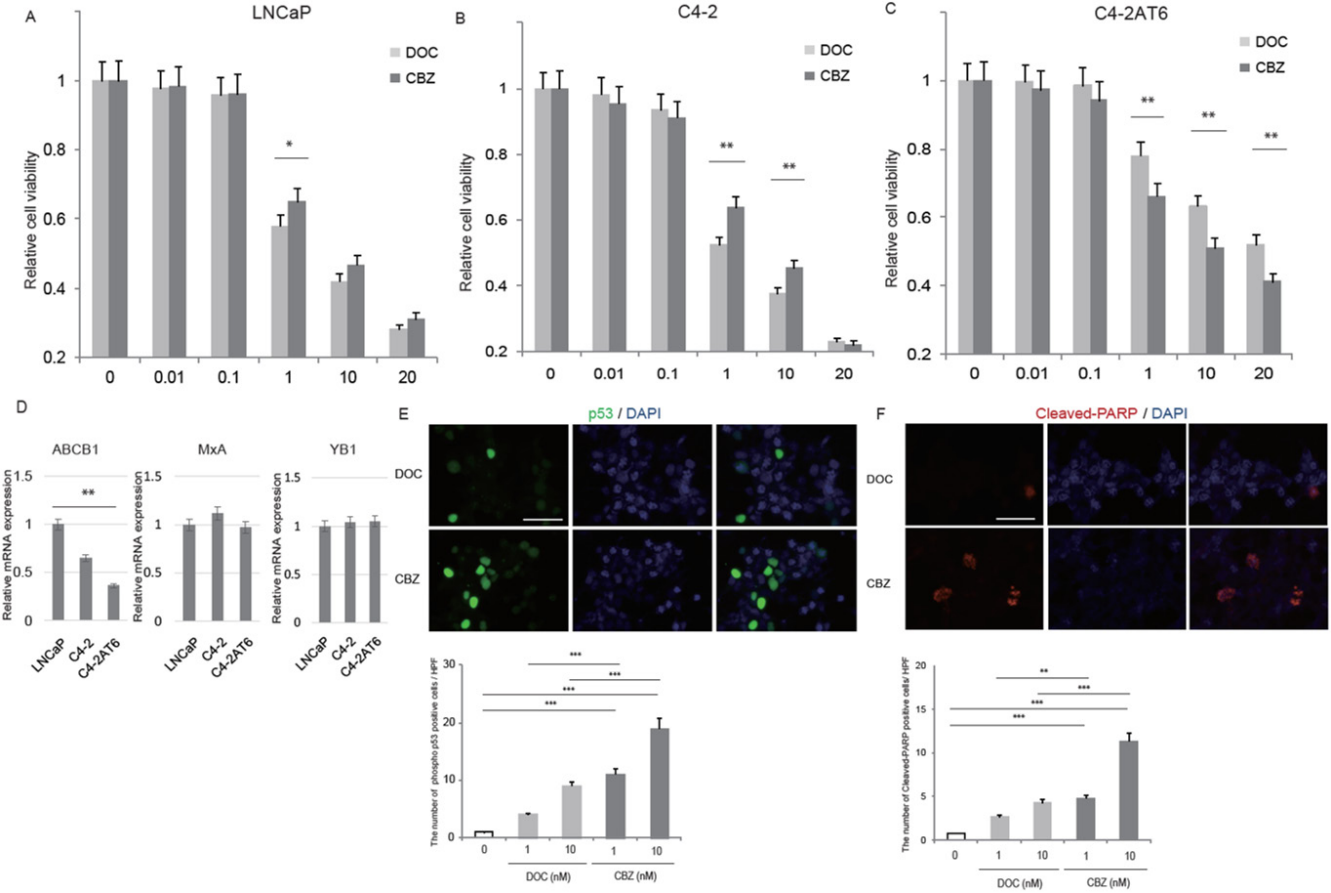
Cabazitaxel can overcome docetaxel resistance in C4-2AT6 cells Sensitivity of docetaxel and cabazitaxel in LNCaP **(A)**, C4-2 **(B)**, and C4-2AT6 **(C)** C4-2AT6 cells showed significantly higher sensitivity to cabazitaxel than docetaxel. Prostate cancer cells were treated with increasing concentrations of docetaxel or cabazitaxel. The cytotoxic response was measured by WST assay, and cell viability was calculated standardized to untreated controls. ^*^p<0.05, compared to control. ^**^ p<0.01, compared to docetaxel at the same concentration of cabazitaxel. CBZ: cabazitaxel, DOC: docetaxel. **(D)** The mRNA expression of p-glycoprotein (ABCB1), Myxovirus resistanse A (MxA), and Y-box binding protein-1 (YB-1) in LNCaP, C4-2, and C4-2AT6 cells. **(E)** Immunocytostaining showed cabazitaxel induced significantly higher expression of phosphorylated p53 in C4-2AT6 cells than docetaxel. **(F)** Elevated expression of the cleaved-PARP in C4-2AT6 cells after treatment with cabazitaxel, although docetaxel alone induced a little elevation of cleaved-PARP.

We have previously reported a useful experimental model of human CRPC [20–23]. Briefly, we cultured PTEN-null, androgen receptor (AR) positive, PSA producing CRPC cell line C4-2 for more than 6 months under androgen ablation conditions and named it C4-2AT6. These cells harbor the following characteristics: androgen independency, aggressive angiogenic properties. Such characteristics are thought to reflect those of patients with CRPC [20–24]. We demonstrated that C4-2AT6 cells showed significantly higher resistance to docetaxel treatment than C4-2 cells *in vivo*, as well as *in vitro*[22, 24]. In this study, we asked whether LNCaP, C4-2 and C4-2AT6 cells responded differently to the cytotoxic effects of docetaxel and cabazitaxel. LNCaP, C4-2 and C4-2AT6 cells were treated with increasing concentrations of docetaxel or cabazitaxel *in vitro*. As shown in Figure 1A-1C, C4-2AT6 cell, which was docetaxel resistant CRPC cell line, showed significantly higher resistance to cabazitaxel, as well as docetaxel, when compared with LNCaP or C4-2 cells. On the other hand, cell viability assay revealed cabazitaxel showed significantly higher cytotoxic efficacy than docetaxel in C4-2AT6 cells (Figure 1C). Therefore we focused on the change of sensitivity between docetaxel and cabazitaxel in C4-2AT6 cell. Previous reports showed that androgen ablation affected the expression level of p-glycoprotein (ABCB1), Myxovirus resistanse A (MxA), and Y-box binding protein-1 (YB-1) in prostate cancer cell [25–29]. We investigated the expressions of ABCB1, MxA, and YB1 in LNCaP, C4-2, and C4-2AT6 cells (Figure 1D). C4-2AT6 cells showed significantly decreased ABCB1 expression compared with LNCaP cells. There were no significant difference of YB-1 and MxA expression among these cell lines. These results indicated that the expressions of ABCB1, YB-1 or MxA expression were not responsible for the different sensitivity in prostate cancer cells.

C4-2AT6 cells were subjected to apoptosis assay. Induction of apoptosis was confirmed by the detection of phosphorylated p53 and cleaved-PARP, which are inducers of early apoptosis. Docetaxel alone induced a little elevation of phosphorylated p53 (Figure 1E). On the other hand, the expression of the phosphorylated p53 in C4-2AT6 cells were significantly up-regulated after treatment with cabazitaxel (Figure 1E). Elevated expression of the cleaved-PARP in C4-2AT6 cells after treatment with cabazitaxel, although docetaxel alone induced a little elevation of cleaved-PARP (Figure 1F). These results indicated that C4-2AT6 cells showed a marked sensitivity to cabazitaxel than docetaxel leading to the induction of apoptosis. These results indicated that cabazitaxel can reduced the acquired resistance to docetaxel of C4-2AT6 cells.

### Elevated ROS production by treatment with cabazitaxel

**Figure 2:**
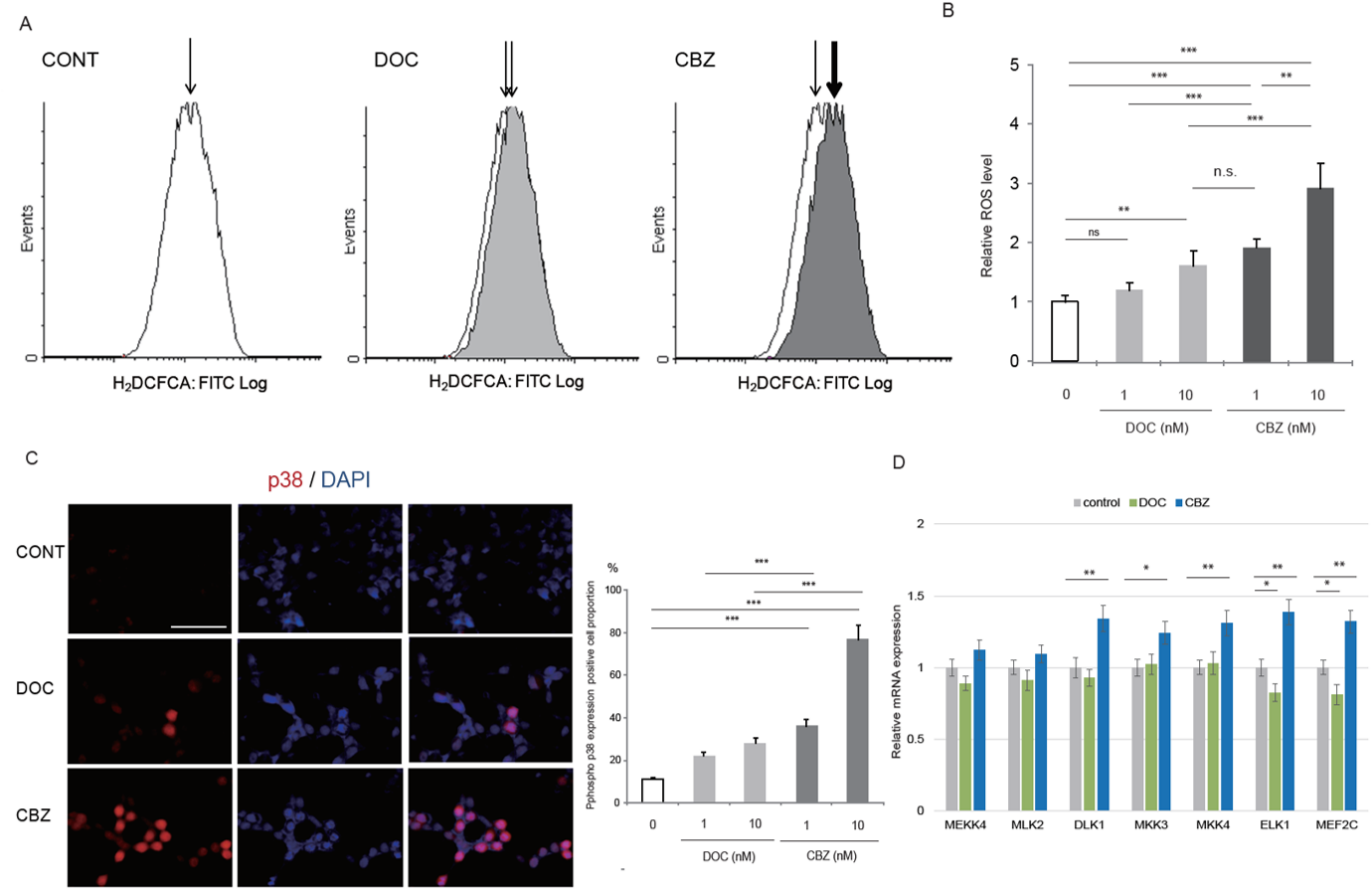
Cabazitaxel induced higher ROS accumulation than docetaxel in CRPC **(A)** Intracellular ROS level in C4-2AT6 cell was determined by fluorogenic marker carboxy-H_2_DCFCA. Fluorescence microscope detected elevated signals of GFP in C4-2AT6 cells treated with cabazitaxel (10 nM) than docetaxel (10 nM). CBZ: cabazitaxel, DOC: docetaxel. **(B)** Carboxy-H_2_DCFCA was quantified by FACS. FACS analysis revealed that treatment with docetaxel slightly increased ROS generation in C4-2AT6 cells. The fluorescent intensities were quantified by FACS. **(C)** Immunocytostaining showed significantly higher level of phosphor-p38^MAPK^ in C4-2AT6 cell when treated with cabazitaxel, compared with docetaxel. **(D)** In C4-2AT6 cells treated with cabazitaxel (CBZ) or docetaxel (DOC), the mRNA expressions of MEKK4 (MAP3K4), MLK2, DLK1, MKK3(MAP2K3), MKK4(MAP2K4), ELK1 and MEF2C which were associated with ROS-mediated cellular signaling. ^*^ ; p< 0.05, ^**^ ; p< 0.01, ^***^ ; p< 0.01, compared with control.

Recent reports demonstrated that ROS production can modulate the cytotoxic effect of taxanes in cancer cells [8, 10, 16, 18]. However, whether cabazitaxel can induce ROS production in CRPC cells or ROS accumulation contributed to the effect of cabazitaxel has not been fully characterized yet. Then, we asked whether cabazitaxel can induce ROS production in C4-2AT6 cells and compared when treated with docetaxel (Figure 2A, 2B). We found cabazitaxel induced intracellular ROS accumulation than docetaxel, shown as elevated signals of green fluorescence. FACS analysis revealed that treatment with docetaxel slightly increased ROS generation in C4-2AT6 cells (Figure 2A, 2B). In contrast, when treated with cabazitaxel in C4-2AT6 cells, ROS generation was significantly induced more than docetaxel (Figure 2A, 2B). To investigate the potential role of cabazitaxel in the cellular response to oxidative stress, we examined the activation of p38^MAPK^, a major downstream target of ROS [30]. Immunoflorescence of p38^MAPK^ analysis revealed that C4-2AT6 cells treated with cabazitaxel showed higher level of phosphor-p38^MAPK^ (Figure 2C). We investigated other potential downstream targets of ROS. We investigated the gene expressions of MEKK4 (MAP3K4), MLK2, DLK1, MKK3(MAP2K3), MKK4(MAP2K4), ELK1 and MEF2C, which were associated with ROS-mediated p38 MAPK cellular signaling. As shown in Fid 2D, among the factors, DLK1, MKK3, MKK4, ELK1, and MEF2C were significantly induced in the treatment of CBZ in C4-2AT6 cells. In contrast, when treated with docetaxel, ELK1 and MEF2C were significantly inhibited. These results indicate cabazitaxel can induce elevated ROS production compared with docetaxel.

### Roles of elevated ROS production by cabazitaxel

**Figure 3:**
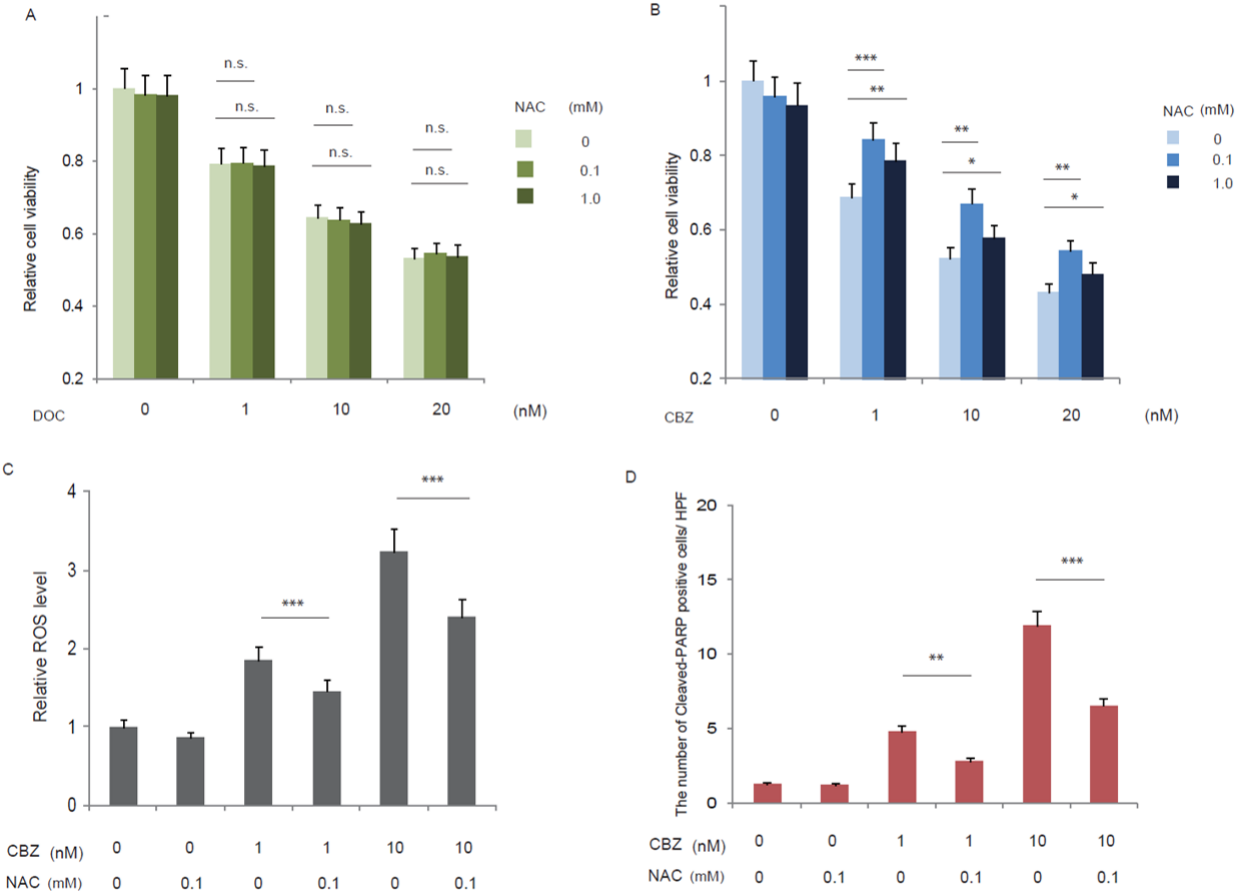
Roles of elevated ROS production by cabazitaxel C4-2AT6 cells were treated with docetaxel **(A)** or cabazitaxel **(B)** in the presence or absence of antioxidant NAC for 24h and evaluated cell survival. NAC eliminated the cytotoxic effect of cabazitaxel in a dose dependent manner. In contrast, NAC didn't eliminated the cytotoxic effect of docetaxel. ^*^ ; p< 0.05, ^**^ ; p< 0.01, ^***^ ; p< 0.01, compared with the viability at the same dose of docetaxel or cabazitaxel alone. The effect of ROS elimination by NAC in C4-2AT6 cells **(C, D)**. Intracellular ROS level in C4-2AT6 cell was determined by fluorogenic marker carboxy-H_2_DCFCA. The fluorescent intensities were quantified by FACS. Elevated expression of the cleaved-PARP after treatment with cabazitaxel was significantly attenuated by NAC (D). ^**^ ; p< 0.01, ^***^ ; p< 0.01, compared with the viability at the same dose of cabazitaxel alone in the presence or absence of antioxidant NAC.

The elevated ROS production by cabazitaxel in C4-2AT6 cells accompanied by higher cytotoxic effect than docetaxel appeared to modulate the effect of cabazitaxel in human CRPC. To address this possibility and to investigate whether cabazitaxel-mediated cell death was caused by the ROS generation induced by cabazitaxel, C4-2AT6 cells were treated with doceatxel (Figure 3A) or cabazitaxel (Figure 3B) in the presence or absence of antioxidant NAC for 24h and evaluated cell survival. We found that NAC didn't reduce the cytotoxic effect of docetaxel. On the other hand, we found that cabazitaxel induced dose-dependent cytotoxicity on C4-2AT6 cells, and NAC reduce the cytotoxic effect in a dose dependent manner (Figure 3B). To investigate the ROS elimination by NAC can modulate the cytotoxic effect, we investigated the ROS level in C4-2AT6 cells treated with cabazitaxel in the presence or absence of antioxidant NAC (Figure 3C). Elevated ROS production was decreased by the treatment with NAC (Figure 3C). Elevated expression of the cleaved-PARP in C4-2AT6 cells after treatment with cabazitaxel was significantly inhibited by NAC (Figure 3D). These results demonstrate that cabazitaxel-induced cell death results from increased ROS generation in C4-2AT6 cells.

### Cabazitaxel induced ROS production through inhibiting antioxidant-Sestrin 3 expression

**Figure 4:**
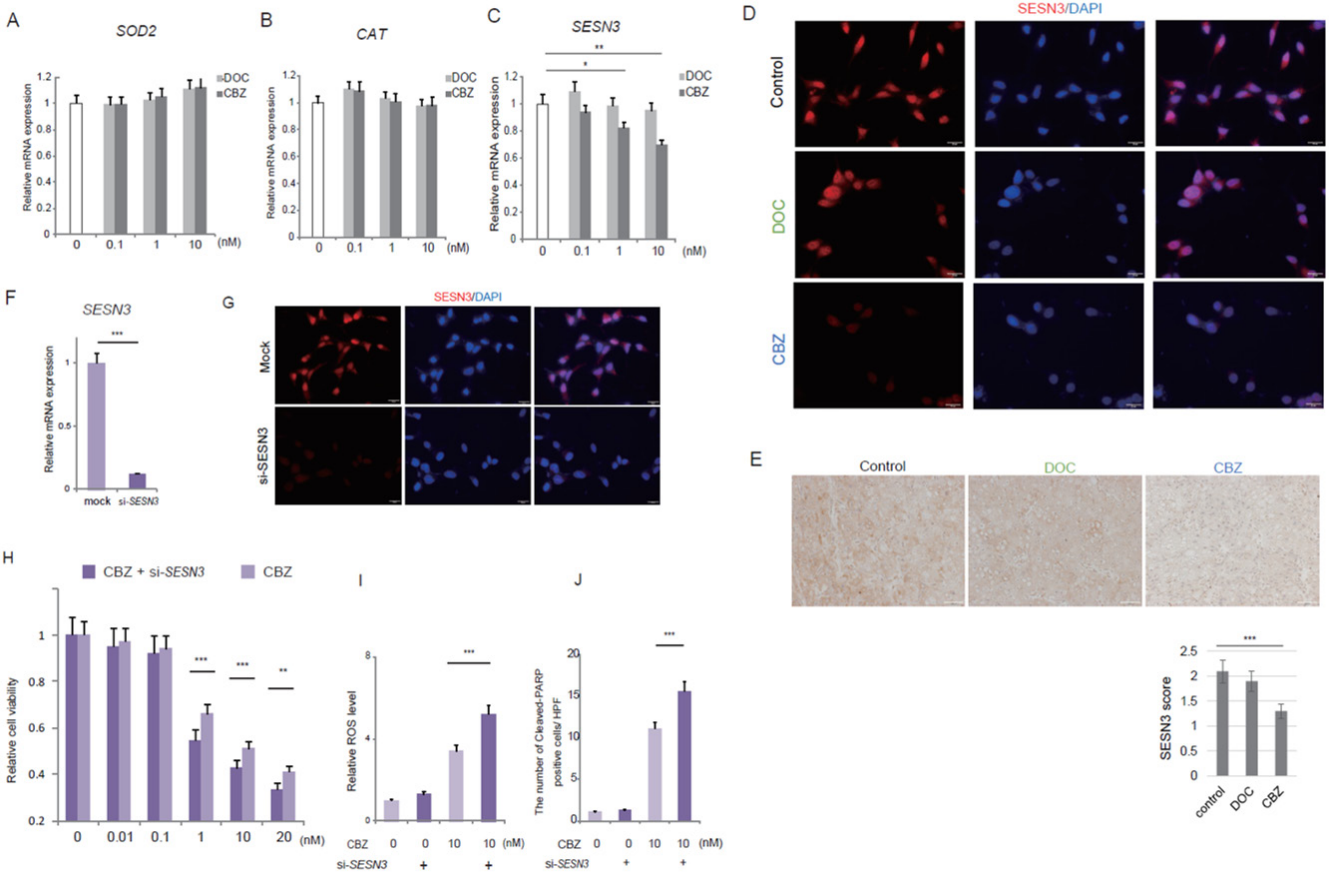
The changes of transcriptional expression of antioxidant enzymes by the treatment with docetaxel or cabazitaxel **(A)** The mRNA expression of manganese superoxide dismutase (MnSOD, SOD2) in C4-2AT6 cells was not changed by the treatment with docetaxel (DOC) nor cabazitaxel (CBZ). **(B)** The mRNA expression of catalase (CAT) was not changed by the treatment with DOC nor CBZ. **(C)** The transcripts of SESN3 were significantly down-regulated by the treatment with cabazitazel, but not by docetaxel. ^*^ ; p< 0.05, ^**^ ; p< 0.01. **(D)** SESN3 expression in C4-2AT6 cell treated with cabazitaxel was significantly inhibited compared with docetaxel-treated cells. **(E)** SESN3 expression *in vivo* in the control, docetaxel-treated or cabazitaxel-treated tumors. ^***^ ; p< 0.001, compared with control tumors. **(F)** Transfection of siRNAs for SESN3 in C4-2AT6 cells. **(G)** Transfection of siRNAs for SESN3 reduced the level of SESN3 expression both in both the nucleus and cytoplasm. **(H)** C4-2AT6 cells were treated with cabazitaxel in the presence of si-SESN3. C4-2AT6 cells with si-SESN3 showed significantly higher sensitivity to cabazitaxel compared with mock-transfection control. ^**^ ; p< 0.01, ^***^ ; p< 0.001, compared with mock-transfection control. **(I)** The effect of ROS production by si-*SESN3* in C4-2AT6 cells. The enhanced cytotoxic effect was accompanied by elevated ROS production. **(J)** The change of expression of the cleaved-PARP in C4-2AT6 cells with si-*SESN3* after treatment with cabazitaxel. ^***^ ; p< 0.001, compared with mock-transfection control

The overall cellular ROS levels are determined by the rates of ROS generation and elimination [8, 10]. Owing to the presence of redox adaptation system in cancer cells, ROS generation induced by cytotoxic agents may not be sufficient to ROS accumulation. Then we asked whether cabazitaxel modulated the expression of antioxidant enzymes. Of the antioxidant enzymes, manganese superoxide dismutase (MnSOD), catalase (CAT) and sestrin 3 (SESN3) are reported to play important roles in ROS detoxication in cancer cells [8, 10, 16–18, 31]. We hypothesized that ROS generation were modulated by the cabazitaxel through inhibiting the expression of these antioxidant enzymes. We investigated the changes of transcriptional expression of MnSOD (Figure 4A), CAT (Figure 4B) and SESN3 (Figure 4C) by the treatment with docetaxel and cabazitaxel. Quatitative real-time PCR analysis revealed that there was no difference between docetaxel-treated and cabazitaxel-treated C4-2AT6 cells in the expression of SOD2 and CAT mRNA (Figure 4A, 4B). On the otherhand, the mRNA expression of SESN3 was significantly inhibited by cabazitaxel in a dose dependent manner, but not by docetaxel (Figure 4C). We investigated the expression and the localization of SESN3-immunofluorescent staining using different concentrations of cabazitaxel (Figure 4D). SESN3 expression in C4-2AT6 cell treated with cabazitaxel was significantly inhibited. We investigated the change of SESN3 expression *in vivo* by using immunohistochemical analysis (Figure 4E). To investigate the expression of SESN3 in the tumor tissue, the mice were assigned to one of three groups: control, docetaxel (5 mg/kg), or cabazitaxel (5 mg/kg). After the tumor had reached a volume of ~200 mm^3^, docetaxel or cabazitaxel was administered i.p. The animals were killed 24 hours later, and the subcutaneous tumors were harvested to investigate the SESN3 expression in the tumor tissue. As shown in Figure 4E, SESN3 expression in the cabazitaxel-treated tumors was significantly decreased compared with docetaxel-treated tumors. These results indicated that cabazitaxel inhibited the expression of one of antioxidant enzyme, SESN3, resulted in reduced ROS elimination leading to elevated ROS generation in C4-2AT6 cell treated with cabazitaxel.

To confirm the possibility and to investigate whether cabazitaxel-mediated cell death was caused by the elevated ROS induced by decreased SESN3 expression, C4-2AT6 cells were treated with cabazitaxel in the presence of siRNAs for SESN3 for 24h and evaluated cell survival. We performed additional experiments to examine the effect of SESN3 knock-down on the sensitivity of C4-2AT6 cells to cabazitaxel. Transfection of siRNAs for SESN3 reduced the level of SESN3 mRNA expression in C4-2AT6 cells by 88.1%, in comparison to that in the cells treated with mock-transfection control (Figure 4E). As shown in Figure 4G, transfection of siRNAs for SESN3 reduced the level of SESN3 expression in both the nucleus and cytoplasm (Figure 4F). We observed significant enhanced cytotoxic effect of si-SESN3 on the C4-2AT6 cells under cabazitaxel treatment compared with mock-transfection control (Figure 4H). The enhanced cytotoxic effect was accompanied by elevated ROS production (Figure 4I) and increased cleaved-PARP expression in C4-2AT6 cells with si-SESN3 (Figure 4J). These results indicate that inhibition of SESN3 expression by cabazitaxel is one of the mechanisms of the effect of cabazitaxel on C4-2AT6: human CRPC model.

## DISCUSSION

In the present study, we described that cabazitaxel showed significantly higher cytotoxic effect in C4-2AT6 cells, accompanied by elevated ROS production through inhibiting antioxidant enzymes; SESN3.

In this study, we found that C4-2AT6 cells showed significantly higher sensitivity to cabazitaxel than docetaxel. Previous reports showed that androgen ablation affected the expression level of p-glycoprotein; ABCB1, MxA or YB1 in prostate cancer cell [25–29]. C4-2AT6 cells showed significantly decreased ABCB1 expression compared with LNCaP or C4-2. Moreover there were no significant difference of MxA or YB1 expresison among these cell lines. These results indicated that ABCB1, MxA or YB1 expression was not responsible for the different sensitivity of docetaxel and cabazitaxel among prostate cancer cells.

Recently, several preclinical studies have suggested a critical role of ROS in cancer therapy [8, 11–15, 19, 32]. ROS regulation can modulate the cytotoxic effect of taxanes in cancer cells [17, 18]. ROS production by cabazitaxel has not characterized yet. We found cabazitaxel induced intracellular ROS accumulation in C4-2AT6 cells than docetaxel. To examine the mechanistic action of ROS generation induced by cabazitaxel, we evaluate the possible contribution of ROS in its cytotoxic effect in the presence or absence of antioxidant NAC. After treatment with cabazitaxel and NAC, C4-2AT6 cells showed marked elevated resistance to cabazitaxel, but not in docetaxel and NAC. These findings indicated that elevated ROS production induced by cabazitaxel treatment accounted for the sensitivity of C4-2AT6 cells. The dysregulation of the balance between ROS production and ROS elimination can contribute to survive or death in cancer cells [10, 31]. Oxidative stress or genotoxic stress can induce Sestrin (SESN) family consisting of SESN1-3. SESNs are stress responsive genes and are activates p53-dependent or independent manner [33, 34]. SESN3 prevents accumulation of ROS and oxidative DNA damages. PI3K/Akt signaling pathways and FOXO families play important role in SESN3 [10, 35]. We previously reported that C4-2AT6 cells showed elevated pAkt expression accompanied docetaxel resistance [22]. These observation support the concept that the correlation between SESN3 and taxanes sensitivity. Our findings constitute direct evidence that the higher sensitivity of human CRPC to cabazitaxel compared to docetaxel involves ROS production through inhibiting the expression of antioxidant enzyme.

ROS have a well-established role in the initiation of cancer through effects on DNA damage, leading to the oncogene activation or loss of tumor suppressor gene function [36]. Recently, several preclinical studies have suggested a critical role of ROS in cancer therapy [8, 10–12, 14, 15, 17–19]. Studies with other types of cancer cells also indicated that ROS can modulate sensitivity to chemotherapeutic agents. These reports suggest that the role of ROS on cancer cells extend beyond their role in DNA damage or genomic instability in early tumorigenesis. Although clinical use of ROS modulators for cancer therapy is thought to be premature, accumulating evidences suggested that ROS modulation may function as multiple and complicated diverse effects in cell survival [37]. Clinical trials for antioxidants in cancer is overwhelmingly negative, suggesting a more careful assessment of antioxidants for cancer therapy are needed including in combination with cytotoxic agents such as cabazitaxel as shown in this study [37].

## MATERIALS AND METHODS

### Reagent

N-acetyl cysteine (NAC) and mouse monoclonal antibody for beta-actin were purchased from Sigma (Atlanta, GA). Rabbit monoclonal antibody for phoshpo-p53 (Ser15), phospho-p38^MAPK^ (D3F9), cleaved PARP were purchased from Cell Signaling Technology (Tokyo, Japan). Rabbit polyclonal antibody for SESN3(bs6100R) was purchased from Bioss (MA, USA). Water soluble tetrazolium (WST) reagents was purchased from Takara Bio Inc. (Kyoto, Japan). Docetaxel were purchased from Wako (Kyoto, Japan). Cabazitaxel were purchased from Toronto Research Chemicals Inc (Ontario, Canada)

### Cell lines and culture

In this study, we used human PCa cell lines: LNCaP, C4-2, C4-2T6cells. C4-2AT6 cells were grown in RPMI-1640 containing 10% charcoal-stripped fetal bovine serum (C-FBS), as previously reported [20–23]. Briefly, C4-2 cells were grown in RPMI-1640 (Invitrogen, Carlsbad, CA) containing 10% charcoal-stripped fetal bovine serum, at 37°C in a humidified 5% CO_2_ atmosphere. These cells were passaged upon reaching confluence during 6 months. We named this cell line as C4-2AT6 (Androgen-ablated treatment for 6 months) [20–23].

### Measurement of intracellular ROS level

In order to measure intracellular ROS level, C4-2AT6 cells were stained with carboxy-2′, 7′-dichlorodihydrofluorescein diacetate (carboxy-H2DCFCA), a reliable fluorogenic marker for ROS in live cells for 30 min, and then harvested according to the manufacturer's protocol (Molecular Probes, NY). The oxidation product of carboxy-H2DCFCA has excitation/emission maxima of approximately 495/529 nm and can be observed using standard filter sets. The fluorescent intensities were quantified using Amnis Flow Sight (EMD Millipore Bioscience, MA) and observed using fluorescence microscope (Olympus, Tokyo) after treatment with docetaxel or cabazitaxel at the same concentration of 10 nM.

### WST assay for cell viability

C4-2AT6 cells were plated in 96-well plates, allowed to attach for 24 h, and then treated with different concentrations of cabazitaxel or docetaxel and/or NAC. At the end of the incubation period, WST reagents were added to each well and incubated for 1 hr. In order to investigate the effect of ROS generation by docetaxel or cabazitaxel, C4-2AT6 cells were treated with docetaxel or cabazitaxel in the presence or absence of antioxidant NAC for 24h and evaluated cell survival. Cell viability was estimated by colorimetry, reading the color intensity in a plate reader at 570 nm. Cell viability was expressed as percent values in comparison with untreated cells.

### Immunocytochemistry

5×10^4^ C4-2AT6 cells were seeded onto glass coverslips and cultivated for 24h before incubation with docetaxel or cabazitaxel at the same concentration of 10 nM for 24 hr. After the treatment, cell were washed with PBS and fixed with 4% paraformaldehyde for 20 min at room temperature. Cells were permeabilized with 0.1% Triton X-100 for 15 min at room tempareture and incubated with 3% BSA. The cells were incubated with anti-phoshpo-p38 MAPK rabbit monoclonal antibody, anti-p53 rabbit monoclonal antibody or cleaved PARP rabbit monoclonal antibody followed by donkey anti-rabbit IgG conjugated to AlexaFluor 555. The results of staining were scored as the product of the percentage of cells stained.

### Real-time quantitative PCR

Total RNA was isolated using RNeasy Mini kit (Qiagen, Hilden, Germany), and the quantity and quality were evaluated by spectrophotometry. Reverse transcription of RNA to cDNA was done using High Capacity cDNA Archive Kit (Applied Biosystems). The reaction mixture (1 μL) was then used as a template in a TaqMan Fast real-time quantitative PCR assay using Taqman Universal PCR Master Mix and the CFX Real-time PCR system (BIO RAD, Tokyo, JAPAN). The primers and TaqMan probe sets (TaqMan Gene Expression Assays) for superoxide dismutase 2: SOD2 (Hs00167309_m1), catalase: CAT (Hs00156308_m1), SESN3 (Hs00376220_m1), ABCB1 (Hs00184500_m1), MxA (Hs00358903_g1), MEKK4(MAP3K4) (Hs00245958_m1), MLK2 (Hs00374249_m1), DLK1 (Hs00171584_m1), MKK3(MAP2K3) (Hs00177127_m1), MKK4 (MAP2K4) (Hs00387426_m1), ELK1 (Hs00901847_m1), MEF2C (Hs00295835_m1), human GAPDH endogenous control (Hs00376220_m1) were purchased from Applied Biosystems. The cycling conditions were 50°C for 10 minutes, 95°C for 10 minutes followed by 40 cycles at 95°C for 15 seconds and at 60°C for 1 minute.

### Small interfering RNA (siRNA)

SESN3 expression was transiently downregulated using the following predesigned duplex siRNA directed against SESN3 (si-*SESN3*; Ambion, Carlsbad, CA, USA). The sense sequences of siRNA for SESN3 were as follows: si-*SESN3*, 5′-GGCUAAUAUCAGUCAACAAtt-3′. C4-2AT6 cells were cultured in antibiotic-free medium overnight at 37°C in 5% CO_2_ and then cells were transiently transfected with 20nmol of si-*SESN3* using Lipofectamine 2000 (Invitrogen Co., Tokyo, Japan). After 4h, siRNA was removed by replacing the culture medium with fresh RPMI 1640 containing 10% charcoal-stripped FBS, and cells were cultured for additional 24-48h. A mock-transfection control was prepared using the transfection reagent only.

### Statistics

Experiments were carried out with three or more replicates and statistical analysis was performed by Student's t test. Experimental values are expressed as mean value ± standard deviation. P values < 0.05 were considered significant.
